# Acute Kidney Injury by Radiographic Contrast Media: Pathogenesis and Prevention

**DOI:** 10.1155/2014/362725

**Published:** 2014-08-14

**Authors:** Michele Andreucci, Teresa Faga, Antonio Pisani, Massimo Sabbatini, Ashour Michael

**Affiliations:** ^1^Nephrology Unit, Department of Health Sciences, “Magna Graecia” University, Campus “Salvatore Venuta”, Viale Europa, Località Germaneto, 88100 Catanzaro, Italy; ^2^Nephology Unit, Department of Public Health, “Federico II” University, Via Pansini no. 5, 80131 Naples, Italy

## Abstract

It is well known that iodinated radiographic contrast media may cause kidney dysfunction, particularly in patients with preexisting renal impairment associated with diabetes. This dysfunction, when severe, will cause acute renal failure (ARF). We may define contrast-induced Acute Kidney Injury (AKI) as ARF occurring within 24–72 hrs after the intravascular injection of iodinated radiographic contrast media that cannot be attributed to other causes. The mechanisms underlying contrast media nephrotoxicity have not been fully elucidated and may be due to several factors, including renal ischaemia, particularly in the renal medulla, the formation of reactive oxygen species (ROS), reduction of nitric oxide (NO) production, and tubular epithelial and vascular endothelial injury. However, contrast-induced AKI can be prevented, but in order to do so, we need to know the risk factors. We have reviewed the risk factors for contrast-induced AKI and measures for its prevention, providing a long list of references enabling readers to deeply evaluate them both.

## 1. Introduction

It is well known that using iodinated radiographic contrast media may cause kidney dysfunction, especially in patients with preexisting renal impairment and in those with diabetes. This dysfunction may range between a slight increase in serum creatinine and severe acute renal failure with anuria [1].

We may define Contrast-Induced Nephropathy (CIN) or contrast-induced Acute Kidney Injury (AKI) as an acute renal failure (ARF) occurring within 24–72 hrs after the intravascular injection of iodinated radiographic contrast media (used to improve the visibility of internal organs and structures in X-ray based imaging techniques such as radiography and computed tomography—CT) that cannot be attributed to other causes. It is therefore an iatrogenic disease which represents the third most common cause of hospital-acquired ARF after surgery and severe hypotension. It is usually a nonoliguric and asymptomatic transient decline in renal function, which is mirrored by an increase of serum creatinine (SCr) by 0.5 mg/dL (or more) or by a 25% (or more) increase in SCr from baseline [2, 3], peaking on the third to fifth day, and returning to baseline within 10–14 days. Since fluctuations in SCr level may occur naturally or in response to acute medical instability [4], it is better to consider, instead of the increase of SCr, the decrease of creatinine clearance (CrCl) calculated from SCr, age, body weight, and gender using either the MDRD (Modification of Diet in Renal Disease) calculation [5] or CKD-EPI (Chronic Kidney Disease Epidemiology Collaboration) equation [6], or the simple Cockcroft-Gault formula: (140 − number years of age) × kg body weight/72/mg/dL of SCr, in females the result × 0.85 [7]. This is called the estimated glomerular filtration rate (eGFR) that allows us to avoid the measurement of CrCl, as derived from 24-hour urine collection, which is a cumbersome, impractical, and inaccurate test.

In some cases, AKI may cause a severe ARF with oliguria (<400 mL/24 hrs), requiring dialysis. In these patients the mortality is high.

The clinical feature and the management of contrast-induced AKI are the same as those for ARF due to other causes [1, 8, 9].

## 2. Incidence

AKI accounts for 12% of all cases of hospital-acquired ARF [10]. It occurs in up to 5% of hospitalized patients who exhibit normal renal function prior to introduction of contrast medium [11].

For outpatients, the risk for AKI when eGFR >45 mL/min per 1.73 m^2^ seems to be very low (about 2%) [12]. In a prospective, observational study of outpatients with mild baseline kidney disease who underwent contrast-enhanced CT, Weisbord and Palevsky [13] observed the occurrence of AKI in less than 1% of outpatients with GFR >45 mL/min per 1.73 m^2^. Thus, AKI is uncommon in patients with normal preexisting renal function; it occurs more frequently in patients with renal impairment particularly if associated with diabetic nephropathy [4].

Bruce et al. [14] carried out a retrospective study analyzing 11,588 patients who underwent CT either without contrast or with a low osmolar contrast medium (iohexol) or an iso-osmolar contrast medium (iodixanol); they observed no significant difference in the incidence of AKI between the iso-osmolar contrast medium and the control groups for all baseline SCr values; the incidence of AKI in the low osmolar contrast medium group was similar to that of the control group up to an SCr level of 1.8 mg/dL; but values of SCr above 1.8 mg/dL were associated with a higher incidence of AKI in the low osmolar contrast medium group [14].

Mitchell et al. [15] sought to define prospectively the incidence of AKI in an unselected, consecutive, heterogeneous population of outpatients who received low osmolar, nonionic contrast (Iopamidol-370, Isovue-370) for a contrast-enhanced CT study of any body region in the emergency department of a large, academic, tertiary care center. The incidence of AKI was 11% (70 out of 633) among the 633 patients enrolled; six of the 70 cases of AKI subsequently developed severe renal failure, five of whom required dialysis or died.

Davenport et al. [16] determined the effect of intravenous (i.v.) low osmolality iodinated contrast material (LOCM) on the development of AKI following CT in patients with stable renal function, stratified by pre-tomography eGFR. It was a retrospective study performed over a 10-year period in 20,242 adult inpatients (10,121 untreated and 10,121 treated with i.v. iodinated contrast media) with sufficient SCr data. They concluded that i.v. LOCM is a risk factor for nephrotoxicity in patients with a stable eGFR <30 mL/min/1.73 m^2^; there was a trend toward significance at eGFR of 30–44 mL/min/1.73 m^2^. No nephrotoxicity was observed in patients with a pre-tomography eGFR ≥45 mL/min/1.73 m^2^. Thus, according to these authors, i.v. LOCM is a nephrotoxic risk factor, but not in patients with a stable SCr level <1.5 mg/dL or eGFR ≥45 mL/min/1.73 m^2^ [17].

In a recent retrospective study on 53,439 patients in whom SCr was regularly checked, McDonald et al. [18] determined the effect of i.v. iodinated contrast material exposure to the incidence of AKI: the incidence of AKI was not significantly different in contrast media group compared to control group. In a systematic review and meta-analysis of controlled studies by the same group examining the incidence of AKI in patients exposed to i.v. contrast medium compared with patients undergoing an imaging examination without contrast medium (control group), the incidence of AKI, dialysis, and death was similar between the contrast medium group and control group. This pattern was observed regardless of i.v. contrast medium type, diagnostic criteria for AKI, or whether patients had diabetes mellitus or renal insufficiency [19].

Rudnick and Feldman [20] have evaluated whether AKI is causally related to mortality and to what extent could mortality in patients undergoing contrast procedures be reduced by preventing AKI. A review of observational studies and clinical trials allowed the conclusion that the deaths of some patients with AKI are complicated by factors that cannot be directly related to the use of contrast media, such as liver disease, sepsis, respiratory failure, and bleeding. However, it is plausible that AKI contributes to cardiovascular causes of death in patients with AKI.

In a 3-year retrospective study in an intensive care unit (ICU), in which 299 patients undergoing a contrast media-enhanced CT scan in whom changes in SCr between baseline and its maximum value over the 96 hours after contrast media injection were recorded, the incidence of AKI was 14%. The need for renal replacement therapy and ICU mortality were significantly higher in cases of AKI [21].

According to Solomon [12] among all procedures utilizing contrast media for diagnostic or therapeutic purposes, coronary angiography and percutaneous coronary interventions (PCI) are associated with the highest rates of AKI [3] mainly related to the intra-arterial injection and to the high dosage of the contrast necessary, and also to the type of patients who have advanced age, one or more comorbid conditions, and more advanced vascular disease, hypertension, and diabetes [22].

Solomon et al. [23] have studied in 294 patients, with follow-up of at least 1 year after contrast exposure, the relationship of AKI to long-term adverse events, such as death, stroke, myocardial infarction, end-stage kidney disease, percutaneous coronary revascularization, coronary artery bypass graft surgery, cardiac arrest, development of congestive heart failure or pulmonary edema, and the need for permanent pacing. The rate of long-term adverse events was higher in individuals with AKI. A reduction in the incidence of AKI and long-term adverse events was observed in regression analyses to adjust for possible known confounders. This supports the view that AKI is causally related to long-term adverse events rates.

Permanent severe renal failure requiring dialysis occurs in 10% of patients with preexisting renal failure who develop further reduction in renal function after coronary angiography [24], or in <1% of all patients undergoing PCI using contrast agents [25].

## 3. Pathogenesis

**Figure 1:**
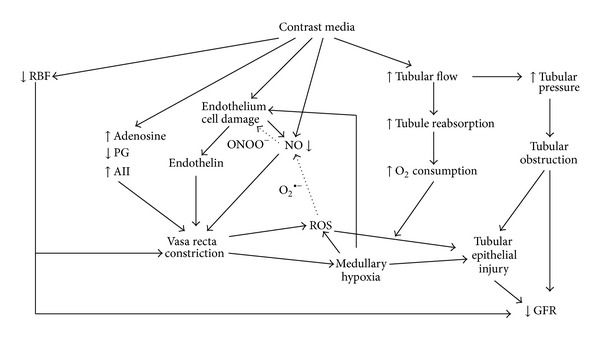
The complex mechanisms that lead to radiocontrast-associated decline of GFR. The dotted arrows indicate the reaction of the reactive oxygen species (ROS) (superoxide anions: O_2_^∙−^) with nitric oxide (NO) that not only causes a reduction in NO levels but also leads to the formation of peroxynitrite anion (ONOO^−^), a potent oxidant that causes cell injury.

The mechanisms underlying contrast media nephrotoxicity have not been fully elucidated and may be due to several factors (Figure 1). When iodinated radiographic contrast media are injected intravenously or intra-arterially, they pass from the vascular compartment through capillaries into the extracellular space. They are eliminated almost entirely by glomerular filtration, concentrated in the renal tubular lumen by water tubular reabsorption, thereby visualizing the urinary tract [1].

The i.v. injection of radiographic contrast medium causes an initial increase in renal blood flow that is then followed by a more prolonged decrease in blood flow and accompanied by a decrease in glomerular filtration rate (GFR), while the extrarenal vessels show transient vasoconstriction followed by a decrease in vascular peripheral resistances. The result will be a renal ischaemia, particularly in the medulla [26, 27]. Oxygen delivery to the outer renal medulla is poor even under normal physiologic conditions because of the distance of the outer renal medulla from the descending* vasa recta*. Thus, the ischemia will be more severe in the outer renal medulla. Medullary ischemia is made more severe by an increase in oxygen consumption (due to increase in tubular reabsorption), an increase of intratubular pressure secondary to contrast-induced diuresis, increased urinary viscosity, and tubular obstruction, all frequently associated with dehydration and decrease in the effective intravascular volume [10].

*In vitro* experiments on the effect of contrast media on arteries obtained from different animal species showed different responses with respect to contraction/dilatation depending on the type of vessels and animal species; in these studies the contrast medium was not applied intraluminally [28].

However, in one study by Sendeski et al. [29], specimens of outer medullary descending* vasa recta* were isolated from rats and microperfused intraluminally with a buffered solution containing iodixanol, with an iodine concentration of 23 mg per milliliter to simulate the usual dosage utilized in examinations in humans. The purpose was to study whether this contrast medium modifies outer medullary descending* vasa recta* vasoreactivity and nitric oxide (NO) production. The authors demonstrated that iodixanol directly constricts the descending* vasa recta* (52% reduction of their luminal diameter) by reducing NO and significantly increases the vasoconstrictor response to angiotensin II, thereby causing severe local hypoxia. The authors conclude that iodixanol in doses typically used for coronary interventions constricts medullary descending* vasa recta*, intensifies angiotensin II-induced constriction, and reduces bioavailability of NO.

Hypoxia may lead to the formation of reactive oxygen species (ROS) [30, 31]. Generated during contrast-induced renal parenchymal hypoxia, ROS may exert direct tubular and vascular endothelial injury and might further intensify renal parenchymal hypoxia by virtue of endothelial dysfunction and dysregulation of tubular transport [32, 33]. The decrease in NO is believed to be due to its reaction with ROS in particular superoxide [28, 34]. This reaction may lead to the formation of the more powerful oxidant peroxynitrite [35] that may be more detrimental. Myers et al. [36] have demonstrated, by* in vivo* experiments in rats, that the decrease in cortical and medullary microvascular blood flow induced by a contrast medium is partly accounted for by the downregulation of endogenous renal cortical and medullary NO synthesis. Sendeski et al. [29] have demonstrated that the superoxide dismutase mimetic Tempol reduced iodixanol-induced vasoconstriction, thereby supporting the role of ROS generated during contrast media administration in medullary descending* vasa recta* vasoconstriction. More recently Pisani et al. [37] have demonstrated that a recombinant manganese superoxide dismutase administered* in vivo* to rats undergoing diatrizoate treatment was able to reduce renal oxidative stress, thereby preventing the reduction of GFR and the renal histologic damage that follows contrast media administration.

The toxicity caused by specific properties of contrast media, such as osmolality, viscosity, and ionic strength, can be differentiated from the cytotoxicity common to all contrast media in studies using cell culture, isolated blood vessels, and isolated tubules; contrast media, in fact, possess a cytotoxicity that is probably caused by iodine and leads to apoptosis and cell death of both endothelial and tubular cells [28].

Thus, the decrease in NO in the* vasa recta* may not be totally accounted for by increased ROS production, as damage to endothelial cells (including apoptosis) may be another important factor; the decreased NO production in descending* vasa recta*, in fact, is partly due to a loss of endothelial cell viability caused by contrast media [28]. Endothelial damage, including nuclear protrusion, cell shrinkage, fenestration of the endothelial layer, and formation of microvilli (“blebbing”) on the cell membrane, and cellular apoptosis have been observed by scanning electron microscopy [38]. Endothelial damage may also release endothelin and hence lead to vasoconstriction. Heyman et al. [39] have in fact demonstrated that i.v. administration of contrast media in rats induced an increase in plasma concentration of endothelin and that contrast media stimulated endothelin release from cultured bovine endothelial cells, suggesting a direct effect of ionic and nonionic agents on vascular endothelium. Reduced levels of prostaglandins have also been suggested to predispose to AKI [40].

In addition to endothelial damage (the endothelial cells are the first to come in contact with intravenously injected contrast agents), contrast media cause damage also on epithelial tubular cells [41]. In fact, the contrast media are filtered by glomeruli and are concentrated inside the renal tubules, exposing the renal tubular cells to even worse direct damage. Direct tubular epithelial cell toxicity by contrast media has been observed in studies of isolated tubule segments and cultured cells substantiated by disruption of cell integrity and apoptosis. The cell damage may be aggravated by factors such as tissue hypoperfusion and hypoxia, by properties of contrast media, such as ionic strength, high osmolarity, and/or viscosity, and by clinically unfavourable conditions, such as preexisting renal impairment particularly secondary to diabetic nephropathy, salt depletion and dehydration, congestive heart failure, and concurrent use of nephrotoxic drugs [1, 28, 42, 43]. The biochemical changes underlying the epithelial damage have been extended to study changes in major intracellular signalling pathways involved in cell survival, death, and inflammation [31, 44–51]* in vitro* in cultured renal tubular cells [52]. Studies in animals and* in vitro* studies suggest that iodinated contrast media can directly induce caspase-mediated apoptosis of renal tubular cells. It seems that contrast-induced apoptosis is due to the activation of shock proteins and the concurrent inhibition of cytoprotective enzymes and prostaglandins [53, 54].

## 4. Risk for Development of AKI

The identification of conditions that represent the risks for the development of AKI is of major importance in the prevention of AKI.

According to the European Society of Urogenital Radiology the real risks for AKI are represented by preexisting renal impairment, particularly when secondary to diabetic nephropathy, by salt depletion and dehydration, by congestive heart failure, by advanced age (>70 years), and by the concurrent use of nephrotoxic drugs [2].

Hereafter we discuss the different risk factors.

### 4.1. Preexisting Impairment of Renal Function

The presence of renal insufficiency, irrespective of its cause, represents the main risk condition. The lower the eGFR is, the greater the risk of AKI following the administration of contrast media will be. According to Mehran and Nikolsky [3] an eGFR of 60 mL/min/1.73 m^2^ is a reliable cutoff point for identifying patients at high risk for the development of AKI, the incidence of which, in patients with underlying chronic renal failure (CRF), ranges from 14.8 to 55%. In a recent retrospective observational in-hospital study in 1160 patients with or without chronic kidney disease (eGFR ≥ 60 mL/min/1.73 m^2^), however, Neyra et al. [55] have observed that AKI occurred with similar frequency, following coronary angiography, in both patients with and without chronic kidney disease (eGFR ≥ 60 mL/min/1.73 m^2^).

### 4.2. Diabetes Mellitus

An important risk factor is diabetes mellitus, particularly when associated with renal insufficiency [56].

In a study by Manske et al. [57] 59 insulin-dependent diabetics with a mean SCr level of 5.9 mg/dL underwent coronary angiography as part of a pretransplant evaluation; 24 azotemic diabetics undergoing inpatient evaluation not including angiography for transplantation formed the control group. Contrast-induced AKI (defined as an SCr increase of greater than 25% when measured 48 hours after radiocontrast exposure) occurred in 50% of patients and none in controls. The authors conclude that azotemic patients with diabetes are at high risk of developing AKI (usually reversible but requiring short-term dialysis in some patients) even when less than 100 mL of radiocontrast agent are used; they suggest using less than 30 mL of radiocontrast agent to minimize renal damage. According to Mehran and Nikolsky [3] at any given degree of baseline GFR, diabetes doubles the risk of developing AKI compared with nondiabetic patients [58]. The incidence of AKI in diabetic patients varies from 5.7 to 29.4%. The administration of iodinated radiocontrast media to diabetics acutely reduces renal parenchymal oxygenation, a reduction that is most prominent in the renal medulla, since it already functions at low oxygen tension [58]. The biologically active endothelins are produced by proteolysis of the precursor preproendothelins under the action of endothelin-converting enzyme that plays a key role in increasing circulating and renal endothelin levels found both in diabetes and after exposure to contrast agents. This may explain the particular susceptibility of diabetic patients to contrast media [58].

The increased incidence of AKI in diabetic patients has also been attributed to hypersensitivity of renal vessels of diabetics to adenosine, a vasoconstrictive agent, since experimental studies have shown increased adenosine-induced vasoconstriction in the kidneys of diabetic animals and the administration of adenosine receptor antagonists reduces the risk of development of contrast-induced AKI in both diabetic and nondiabetic patients [59].

It has been demonstrated that, in patients with diabetes, hypercholesterolemia is the strongest predictor of AKI [60].

Despite the evidence mentioned, most authors do not regard the presence of diabetes mellitus in the absence of renal failure as a risk factor for AKI [61]. In a prospective observational study Morabito et al. [62] have evaluated the incidence of contrast-induced AKI in all unselected patients who underwent elective or emergency coronary angiography or PCI in their department throughout a period of 11 months. They observed a 5.1% incidence of AKI. In diabetic patients with preserved renal function and without other risk factors, the rate of AKI was comparable to that of a nondiabetic population, while clinically important AKI occurred in diabetic patients with underlying chronic renal disease [1].

### 4.3. Concomitant Use of Other Drugs

Radiocontrast media are medical drugs used for diagnostic purpose. The concomitant use of other drugs may represent a risk factor for contrast-induced AKI. This is undoubtedly the case when using nephrotoxic drugs, such as aminoglycosides (which have a direct nephrotoxic effect), amphotericin (causes distal tubule dysfunction, impaired urine concentration and potassium and magnesium wasting), cyclosporin A (a direct cellular toxin which impairs lysosome function in both proximal and distal tubules and evokes tubulo-interstitial changes), cisplatin (attaches to sulphhydryl groups which are essential for proper enzyme function) [63].

Also the concomitant use of nonsteroidal anti-inflammatory drugs represents an important risk factor because of their inhibition of the vasodilatory prostaglandins biosynthesis. According to Morcos [64], in fact, the damaging effect of contrast media on the kidney partly involves the osmolality-dependent activation of the tubuloglomerular feedback mechanism and the modulation of the intrarenal production of vasoactive mediators such as prostaglandins, NO, endothelin, and adenosine. Thus, reduction in the synthesis of the endogenous vasodilator prostaglandins (as occurring following the use of nonsteroidal anti-inflammatory drugs) will increase the nephrotoxicity of contrast media.

The concomitant use of angiotensin-converting enzyme inhibitors (ACEIs) or angiotensin receptor blockers (ARBs) may also represent a risk factor, at least according to some authors. There are, however, conflicting opinions on this point.

Some authors have described protective effects. Thus, considering the possible role of medullary ischaemia mediated by renin angiotensin system in genesis of contrast-induced AKI, Gupta et al. [65] investigated the role of the ACEI captopril in preventing AKI. The rationale was that angiotensin II is a main effector peptide in the renin-angiotensin system and plays a very important role in controlling renal homeostasis as a vasoconstrictor. Thus, captopril might prevent AKI by reducing the increase in angiotensin II. Seventy-one patients with diabetes mellitus undergoing coronary angiography were included in the study. Patients received captopril in a dose of 25 mg thrice a day for three days, starting one hour prior to angiography, while the patients in the control group underwent angiography without receiving captopril. AKI developed in 29% of the control group; the administration of captopril reduced the risk of development of contrast-induced AKI by 79%. The authors concluded that captopril offers protection against development of contrast-induced AKI.

Similarly, in an experimental study in Sprague-Dawley rats, Duan et al. [66] administered telmisartan to confirm its protective role against nephrotoxicity induced by contrast media. Glycerin was given to all rats to induce renal injury. Diatrizoate, a high-osmolar contrast medium (HOCM), or iohexol, a low-osmolar contrast medium (LOCM) (10 mL/kg b.w., 300 mg I/mL), was given through a caudal vein. In diatrizoate-injected rats, SCr level was increased (*P* < 0.001). Both HOCM (diatrizoate) and LOCM (iohexol) caused renal tubular cell apoptosis in the kidneys damaged by glycerin. The renal caspase-3 activity and angiotensin II levels in HOCM and LOCM groups were higher than those in glycerol control group (*P* < 0.001). The renal injury was also assessed by histology. Telmisartan protected the renal tissue from nephrotoxicity induced by contrast media.

In contrast, many have suggested that patients with chronic renal disease under treatment with ACEIs or ARBs are at higher risk for developing AKI particularly in the elderly. Thus, Cirit et al. [67] have evaluated the influence of chronic ACEIs administration on the development of contrast-induced AKI in patients undergoing coronary angiography. The 230 patients with renal insufficiency and age ≥65 years were divided into two groups: 109 users of ACEI, ACE inhibitor group, 121 nonusers, control group: AKI occurred in 17 patients (15.6%) of the ACEI group and 7 patients (5.8%) of the control group (*P* = 0.015). They conclude that chronic ACEI administration is a risk for developing AKI in elderly patients with renal insufficiency.

Kiski et al. [68] have performed a prospective, single-centre study to compare different treatments for AKI prevention; 412 patients were included in the study, 269 (65.3%) of whom were taking ACEI (*n* = 236) or ARBs (*n* = 33). The occurrence of AKI within 72 h was significantly higher in patients treated with ACEI or ARBs (11.9 versus 4.2%, *P* = 0.006).

In a retrospective study of Rim et al. [69] among 11,447 patients receiving coronary angiography or PCI, 1,322 were receiving either ACEI or ARBs. ACEI/ARBs users showed an increased incidence of contrast-induced AKI compared to nonusers: 11.4% versus 6.3% (*P* < 0.001).

A single-center retrospective case-control study was conducted by Umruddin et al. [70] on a total of 201 patients who were exposed to nonionic radiocontrast agents for coronary angiography, to evaluate the influence of ACEI and ARBs use in the etiology of AKI. They identified patients who met the criteria for AKI (a rise in SCr of more than 25% from the baseline within 48 hours of radiocontrast agent exposure and the absence of another cause) (AKI group); from the same list they also identified an age-, sex-, and baseline SCr-matched control group who did not meet the criteria for AKI (control group). They found that 56 patients (58.3%) out of 96 of the AKI group were on chronic ACEI or ARBs therapy, while the control group had only 36 (34.3%) out of 105 patients (*P* < 0.001). They concluded that the use of ACEI and ARBs is an independent risk factor for developing AKI.

Some authors suggest discontinuing the use of ACEIs and ARBs 48 hours prior to exposure to radiocontrast agents, especially in patients with multiple risk factors [70, 71].

Others, however, believe that withholding ACEIs and ARBs 24 h before coronary angiography does not influence the incidence of AKI in stable patients with CRF. Thus, Rosenstock et al. [72] undertook a randomized trial to evaluate the effect of withdrawing ACEIs or ARBs 24 h prior to coronary angiography on the incidence of AKI associated with coronary angiography. The 220 patients with CRF, stages 3-4 (eGFR 15–60 mL/min/1.73 m^2^), on ACEI or ARB therapy were randomized before angiography to either ACEI/ARB continuation group or discontinuation group. There was no statistically significant difference in the incidence of AKI. The authors concluded that withholding ACEIs and ARBs 24 h before coronary angiography does not appear to influence the incidence of AKI in stable patients with CRF stages 3-4.

### 4.4. Reduction of Effective Circulating Blood Volume

Dehydration and salt depletion secondary to abnormal fluid losses (gastrointestinal, renal, or dermal losses) associated with insufficient salt intake represent a predisposing condition to AKI by radiographic contrast media, as it is predisposing to any form of ARF [73]. But the reduction of “effective” circulating blood volume is also a risk factor to any form of ARF [8] and in particular to AKI by contrast media.

The “effective” circulating blood volume may be defined as the relative fullness of the arterial tree as determined by cardiac output, peripheral vascular resistance, and total blood volume; it is usually reduced in congestive heart failure (because of reduced cardiac output), in cirrhosis with ascites (because of reduced peripheral resistance), and in nephrotic syndrome (because of reduced blood volume secondary to protein losses) [8].

A reduction of “effective” circulating blood volume may be due to congestive heart failure, compromised left ventricle systolic performance, prolonged hypotension, or liver cirrhosis or nephritic syndrome. Under such circumstances renal vasoconstriction induced by adenosine is accentuated thereby making renal ischemia more severe.

### 4.5. Multiple Myeloma

Multiple myeloma is a malignancy with clinical severity and variable survival time. AKI by contrast media was described for the first time in 1954 in a patient with multiple myeloma receiving intravenous pyelography [74]. Many radiologists have withheld contrast agents from all patients with myeloma, afraid to induce AKI following iodinated radiographic contrast medium use. Early articles, in fact, linked the intravenous administration of contrast agents with the development of renal failure in patients with multiple myeloma, leading to the conclusion that iodinated radiographic contrast media are contraindicated in patients with myeloma [75–79].

In a recent retrospective clinical study Pahade et al. [80] examined the risk of AKI in patients with multiple myeloma following nonionic iodinated contrast media injection during CT. Their retrospective review of medical records identified patients with a diagnosis of myeloma who underwent a contrast-enhanced CT examination of the chest, abdomen, or pelvis. Their search yielded a total of 56 eligible myeloma patients (24 women and 32 men) who underwent a total of 103 CT examinations; the average age was 65 years (range 37–93 years). AKI was defined by an increase in SCr, after the examination, of 25% or more, or of 0.5 mg/dL or more, compared with its level before the examination, both within 48 hours and within 7 days of contrast-enhanced CT. The results showed a 5% incidence of AKI using the 48-hour definition. On the basis of their results, the authors concluded that the incidence of AKI following contrast media administration in patients with multiple myeloma with a normal SCr is low and correlates with *β*_2_-microglobulin levels; thus, the administration of contrast agents in these patients is relatively safe. The serum level of *β*_2_-microglobulin increases with higher tumor burden and with diminished renal function. In their study the mean *β*_2_-microglobulin level has shown a statistically significant association with the development of AKI. According to the authors, a review of *β*_2_-microglobulin serum levels may be beneficial before administering the contrast agent to patients with myeloma, because it likely serves as a marker of patients who are at a higher risk of developing AKI. They suggested a threshold value of less than 2.8 mg/L of *β*_2_-microglobulin serum level for essentially eliminating the risk of AKI [80].

The assessment of Bence Jones proteinuria is unnecessary for evaluating the risk of kidney failure in patients with multiple myeloma, since this test cannot be considered a surrogate biomarker of kidney function [81].

We may conclude that multiple myeloma* per se* cannot be considered a main risk factor for developing AKI following intravascular administration of iodinated contrast media. The risk, however, becomes important when associated with comorbidities such as CRF, diabetes, hypercalcemia, dehydration, and use of nephrotoxic drugs [81].

### 4.6. Osmolality and Viscosity of Contrast Media

Osmolality of contrast media compared with the osmolality of plasma seems to play an important role in nephrotoxicity. Contrast media usually have high viscosity and greater osmolality (more molecules per kilogram of water) than plasma. Ionicity is the characteristic of a molecule to break up into a cation and an anion, resulting in more molecules per kilogram of water and thus increasing osmolality. Nonionic agents not having this property are less osmolar. The osmotoxic effect of contrast agents is described in terms of the ratio of iodine atoms to dissolved particles: the higher the ratio, the better the attenuation of X rays [10, 82].

Ionic high-osmolar contrast media (HOCM, e.g., diatrizoate, 1500 to 1800 mOsm/kg, i.e., 5–8 times the osmolality of plasma) have a ratio of 1.5 : 1, nonionic low-osmolar contrast media (LOCM, e.g., iohexol, 600 to 850 mOsm/kg, i.e., 2-3 times the osmolality of plasma) have a ratio of 3 : 1, and nonionic iso-osmolar contrast media (IOCM, e.g., iodixanol, approximately 290 mOsm/kg, i.e., same osmolality as plasma) have a ratio of 6 : 1 [83].

Adverse reactions to contrast media range from 5% to 12% for HOCM and from 1% to 3% for LOCM. It has been shown that LOCM rather than HOCM are beneficial in the prevention of contrast-induced AKI to patients with preexisting renal failure [82, 84–86]. Iodixanol (IOCM) seems less nephrotoxic than iohexol (LOCM) [82, 87], at least in patients with intra-arterial administration of the drug and renal insufficiency [88, 89]. But recent studies and meta-analyses have found no significant difference in the rates of AKI between IOCM and LOCM [88–93].

In addition to the osmolality of iodinated contrast media, their viscosity is very important, indeed; while the osmolality of a given contrast medium solution, in fact, increases only linearly with the molar concentration, the viscosity increases exponentially [94]. Thus, the low osmolality achieved with the IOCM came at the price of considerably increased viscosity; at comparable iodine concentration and X-ray attenuation nonionic dimer IOCM have about twice the viscosity of nonionic monomer LOCM [95]. The higher viscosity of nonionic dimer IOCM probably relies on a number of the compounds' features including the molecules' shape and the flexibility of the bridge between the two benzene nuclei [96].

Most of the water filtered by the glomerulus is reabsorbed along the length of the renal tubule, thereby causing considerable concentration of the contrast medium within the tubule itself. This results in a progressive increase in tubular fluid osmolality and, due to the exponential concentration-viscosity relationship, an overproportional increase in tubular fluid viscosity as well as in the urine viscosity [94].

Since the fluid flow rate through a tube increases with the pressure gradient and decreases with the flow resistance and since the resistance increases proportionally to fluid viscosity, the increased viscosity caused by a contrast medium increases the intratubular pressure [97]. This causes a decrease in glomerular filtration and contributes to renal medullary hypoperfusion and hypoxia since circular distension of the tubules results in compression of medullary vessels such as the* vasa recta* [94]. On the other hand, the increased flow resistance markedly slows down the tubular flow rate, thereby increasing the contact time of cytotoxic contrast medium with tubular epithelial cells and consequently increasing their damaging effect.

In case of dehydration angiotensin II and vasopressin augment tubular fluid reabsorption, which further increases the tubular concentration of the contrast medium, and, due to the concentration-viscosity relationship, overproportionally increases tubular fluid and urine viscosity. Accordingly, dehydration and/or volume contraction are major individual risk factors for contrast-induced AKI [94]. Hence, the strong recommendation for hydration of the patients before the exposure to contrast media, particularly in the elderly due to an impaired sensation of thirst [98].

*In vivo* studies that directly compared urine viscosities following LOCM versus IOCM administration in dogs and rats demonstrated a larger increase in urine viscosity following IOCM [94, 99–102]. The LOCM causes an increase of tubular fluid viscosity; but the viscosity increase by IOCM is several times larger; the higher the viscosity and the lower the osmolality, the longer the cells exposed to contrast media and the more they are injured [94].

Micropuncture studies in rats found that the IOCM, iotrolan, increased tubular pressure and decreased single nephron GFR much more than HOCM and LOCM did [97, 103].

### 4.7. Use of Large Doses or Multiple Injections of Iodinated Contrast Media

The risk of contrast-induced AKI is dose-dependent; it increases with the volume of contrast medium administered during the procedure and with their multiple injections within 72 hours [104–106]. Larger volumes of contrast agents are used in coronary angiography than in other imaging studies. Therefore, patients who undergo coronary angiography (these patients usually have one or more comorbid conditions) have AKI more frequently than other patients [107, 108].

### 4.8. Route of Administration of Iodinated Contrast Media

Many studies have demonstrated that i.v. contrast media are less risky than intra-arterial contrast media [109–111]. Iodinated radiographic contrast media are more nephrotoxic when given intra-arterially because of the higher acute intrarenal concentration [10, 88], particularly if the arterial injection is suprarenal [112]. It has been demonstrated that, while performing aortography, the closer to the renal arteries the injection of contrast medium occurs, the higher the risk of AKI will be [104].

Dong et al. [88] have performed a study to examine the association between administration route and relative renal safety of iodinated radiographic contrast agents. They searched all published articles indexed in Embase, Medline, and the Cochrane Central Register of Controlled Trials, in the period 1980–2010 and found 11 randomized controlled trials including 2,210 patients with intra-arterial route and 7 including 919 patients with intravenous route of administration. The meta-analysis suggested that administration route may affect the renal safety of different contrast agents. Their results showed that compared with a pool of LOCM, iodixanol (IOCM) was associated with less risk of contrast-induced AKI when administered intra-arterially rather than intravenously.

### 4.9. Advance Age

Advance age, that is, >65 years, represents a predisposing factor to contrast-induced AKI. The reasons for higher AKI risk in the elderly are multifactorial, including age-related changes in renal function (which favours renal sodium and water wasting) [98], the presence of old vessels, of one or more comorbid conditions, such as dehydration, due to impaired sensation of thirst in old subjects, or chronic renal disease particularly if under treatment with ACEIs or ARBs, and the presence of more advanced vascular disease, of coronary artery disease, of longstanding hypertension, and of diabetes.

### 4.10. The Presence of Anemia

Anemia is a risk factor for AKI by contributing to renal ischemia. Nikolsky et al. [113] have studied the relationship between hematocrit and the occurrence of AKI. Of 6,773 consecutive patients treated with PCI, contrast-induced AKI (an increase of ≥25% or ≥0.5 mg/dL in preprocedure SCr, at 48 hours after procedure) occurred in 942 (13.9%) patients. The rates of AKI were the highest (28.8%) in patients who had the lowest level for both baseline eGFR and hematocrit; patients with the lowest eGFR but relatively high baseline hematocrit values had remarkably lower rates of AKI. The authors conclude that correcting the hematocrit before PCI might decrease the rates of contrast-induced AKI.

### 4.11. Sepsis

Sepsis is a risk factor, probably because of direct tubular damage by bacterial toxins and impairment of circulation [63].

### 4.12. The Presence of Transplanted Kidney

Patients with renal transplantation are at a higher risk of contrast-induced AKI due to concomitant use of nephrotoxic drugs, such as cyclosporine, and higher prevalence of diabetes and renal insufficiency. Ahuja et al. [114] have evaluated the safety of iodinated radiographic contrast injections in renal allograft recipients. In a retrospective study they identified 44 patients with functioning renal allograft who underwent different i.v. or intra-arterial contrast studies. Renal function tests were done before and after the contrast study in 35 of these patients, who underwent coronary angiogram in 6 patients, CT scan with intravenous contrast in 11, angiogram for evaluation of peripheral vascular disease in 11, allograft angiogram with angioplasty in 5, pulmonary angiogram in 1, and intravenous pyelogram in 1 patient. The incidence of AKI (≥25% increase in baseline SCr) in the renal allograft recipients was 21.2% (7 of 33 patients). The incidence of AKI was lower 15.3% (4 of 26) in patients who received i.v. hydration compared to 42.8% (3 of 7) in patients who received no prophylaxis prior to radiographic contrast agents.

## 5. Prevention of AKI

It is absolutely necessary to try to prevent contrast-induced AKI. This is even more necessary in high risk patients. The following are useful suggestions for its prevention.

### 5.1. Monitoring Renal Function

Renal function should be monitored in any patient before any radiographic procedure that requires the use of radiographic iodinated contrast agents. SCr should be checked before and after the use of contrast medium. In patients at high risk of AKI, SCr should be checked before and once daily for 5 days after the radiographic procedure [1]. The increase in SCr after the contrast agent administration will indicate nephrotoxicity.

### 5.2. Removal of Nephrotoxic Drugs

Potentially nephrotoxic drugs should be discontinued, whenever possible, before the contrast procedure. This is the case with aminoglycosides, whose direct nephrotoxic effect would potentiate the contrast nephrotoxicity, vancomycin, amphotericin B, cisplatin, and nonsteroidal anti-inflammatory drugs.

In those cases in which aminoglycosides cannot be removed, its dosage should be reduced. Thus, the European Renal Best Practice (ERBP) [115] suggests, for the treatment of infections in patients with normal kidney function in steady state, to administer aminoglycosides as a single dose daily rather than multiple doses, but with monitoring of aminoglycoside blood levels. For amphotericin B, the ERBP recommends that saline loading be implemented in all patients receiving any formulation of amphotericin B [115].

Metformin is a biguanide (dimethylbiguanide) that is used in patients with non-insulin-dependent diabetes mellitus (type II diabetes) as an oral antihyperglycemic medication. Since it stimulates intestinal production of lactic acid, potential harm may happen when renal failure occurs. Approximately 90% of metformin is eliminated via the kidneys in 24 hrs. Thus, renal insufficiency (GFR < 70 mL/min) will lead to its retention in the tissues and to lactic acidosis that can be fatal, since the onset of renal injury after the administration of contrast medium is quite rapid. Thus, the drug has to be discontinued at least 12 hours before the contrast and not be resumed for a minimum of 36 hours after the procedure, or longer if the SCr has not returned to baseline [116].

We have already discussed the controversial opinions on the role of ACEIs and ARBs as potential risk factors for contrast-induced AKI. According to KDIGO guidelines for Acute Kidney Injury Work Group, there is insufficient evidence to recommend discontinuation of these medications prior to contrast administration [117].

### 5.3. The Choice of the Radiographic Contrast Agent

It is very important to choose the least nephrotoxic radiocontrast agent. The LOCM (e.g., iohexol) are less nephrotoxic than HOCM (e.g., diatrizoate). Furthermore, the IOCM (e.g., iodixanol) seem to be less nephrotoxic than the LOCM [1, 10].

A multicenter, randomized, double-blind comparison of iopamidol (LOCM) and iodixanol (IOCM) has been performed by Solomon et al. [91] in patients with chronic kidney disease. The incidence of contrast-induced AKI was not statistically different after the intra-arterial administration of iopamidol or iodixanol to high-risk patients, with or without diabetes mellitus. The authors conclude that iodixanol (IOCM) and iopamidol (LOCM) are iodinated contrast agents of choice to reduce risk of AKI.

### 5.4. The Dosage of the Radiographic Contrast Agent

The lowest dosage possible of the radiographic contrast agent should be used.

High doses of contrast agents are required in PCI. For this procedure, some formulas have been suggested to calculate the dosage that is least dangerous for renal function [1].

1. Cigarroa's formula: 5 mL of contrast per kg b.w./SCr (mg/dL) with maximum acceptable dose of 300 mL for diagnostic coronary arteriography [118].
2. Laskey's formula: volume of contrast to calculated creatinine clearance ratio with a cut-off point of the ratio at 3.7 for PCI; a ratio >3.7 would be associated, following contrast use, with a decrease in CrCl [119]. Recently Gurm et al. [120] have suggested a cut-off point at 2.0: below a ratio of 2.0 AKI would be a rare complication of PCI, but it would increase dramatically at a ratio of 3.0.
3. A new formula seems to be superior and consists of a ratio of grams of iodine to the eGFR; a ratio of 1.42, or even better a ratio of 1.0, would prevent contrast-induced AKI [121].

### 5.5. Adequate Hydration

The crucial preventive measure of contrast-induced AKI is an adequate hydration of the patient [122, 123]. We must abolish the old suggestion to avoid any oral intake starting the day before contrast administration, a measure decided to prevent vomiting and nausea, that was common with high-osmolality contrast agents, and to allow for tracheal intubation in case of any emergency. Undoubtedly the strategy to keep the patient in a fasting state was correct; but many patients and physicians erroneously considered a restriction in fluids in parallel with the restriction in food [122]. This misconception caused patient dehydration before using iodinated contrast media.

Volume supplementation is the cornerstone for the prevention of contrast-induced AKI. According to Mueller [122] an oral or intravenous volume supplementation effectively prevents AKI in low- and moderate-risk patients: 500 mL of water or soft drinks (e.g., tea) orally before and 2,500 mL for 24 hours after contrast administration in order to secure urine output of at least 1 mL/min in a non-dehydrated patient; or i.v. injection of 100 mL/hr of 0.9% saline solution starting 4 hrs before contrast administration and continuing for 24 hrs afterward [124].

High infusion rate or high total fluid volume may result in volume overload and trigger pulmonary edema in patients with predisposing cardiac conditions. In these patients a rather low infusion rate of 1 mL/kg per hour has in general been recommended and used in clinical practice [125]. In high-risk patients adequate hydration may be obtained by i.v. infusion of 0.9% saline at a rate of approximately 1 mL/kg b.w.per hour, beginning 6–12 hours before the procedure and continuing for up to 12–24 hours after the radiographic examination; this may be done only if urine output is appropriate and cardiovascular condition allows it [122].

The rationale for volume supplementation is that hydration causes expansion of intravascular volume, suppression of renin-angiotensin cascade, and consequent reduction of renal vasoconstriction and hypoperfusion. The resulting increase of diuresis will decrease the concentration of contrast material within the tubule lumen and its contact time, thereby diminishing its direct toxicity on tubular epithelium; a higher urine output is associated with a lower incidence of contrast-induced AKI [125].

Some clinical studies and meta-analyses have shown that sodium bicarbonate hydration is superior to sodium chloride [126–132] at least when using LOCM [133].

Thus, Merten et al. [126] treated 119 patients with preexisting renal insufficiency, scheduled mainly for cardiac catheterization, to receive either 154 mEq/l sodium bicarbonate or equimolar sodium chloride, both given as an i.v. bolus (3 mL/kg per hour for 1 hour) immediately before the administration of iopamidol, followed by an infusion at a rate of 1 mL/kg per hour for 6 hours after the procedure. The incidence of AKI (defined as an increase of ≥25% of baseline SCr within 2 days) was lower in the bicarbonate group: 1.7% versus 13.6% (*P* = 0.02). Similarly Masuda et al. [127], using the same protocol of bicarbonate infusion (number: 30) versus saline (number: 29) in 59 patients undergoing an emergency coronary angiography or intervention, found an incidence of AKI of 7% versus 35% (*P* = 0.01).

In a systematic review and meta-analysis using the MEDLINE database, Navaneethan et al. [129] compared the hydration with i.v. sodium bicarbonate with or without N-acetylcysteine versus hydration with normal saline with or without N-acetylcysteine. Sodium bicarbonate significantly decreased the incidence of contrast-induced AKI.

The rationale for using bicarbonate infusion is explained by the fact that any condition (such as acetazolamide administration or sodium bicarbonate infusion) that increases bicarbonate excretion decreases the acidification of urine and medulla. Consequently, this will reduce the production (namely, inhibition of the generation of hydroxyl radicals from H_2_O_2_) and increase the neutralization of oxygen free radicals, thereby protecting the kidney from injury by contrast agents [128, 129, 134].

Other investigators did not find a benefit with sodium bicarbonate hydration versus sodium chloride. Thus, in a study of Brar et al. [135] Medline, EMBASE, Cochrane library, and the Internet were searched for randomized controlled trials comparing hydration between sodium bicarbonate and chloride for the prevention of contrast-induced AKI between 1966 and November 2008. A significant clinical and statistical heterogeneity was observed that was largely explained by trial size. Among the large randomized trials there was no evidence of benefit for hydration with sodium bicarbonate versus sodium chloride (10.7 and 12.5%, resp.) for the prevention of AKI. The authors believe that the benefit of sodium bicarbonate was limited to small trials of lower methodological quality.

Shavit et al. [136] conducted a prospective, single-center trial in 93 patients with CRF, stages III-IV, undergoing cardiac catheterization who received either an infusion of 0.9% sodium chloride and oral N-acetylcysteine (number: 42) or 154 mEq/L sodium bicarbonate (number: 51). They concluded that hydration with sodium bicarbonate is not more effective than hydration with sodium chloride and oral N-acetylcysteine for the prevention of contrast-induced AKI.

Vasheghani-Farahani et al. [137] prospectively enrolled, in a single-center, double-blind, randomized, controlled trial from August 2007 to July 2008, 72 patients undergoing elective coronary angiography with an SCr level ≥1.5 mg/dL, uncontrolled hypertension, compensated severe heart failure, or a history of pulmonary edema; the patients were assigned to either an infusion of sodium bicarbonate plus half saline (*n* = 36) or half saline alone (*n* = 36). The combination therapy of sodium bicarbonate plus half saline did not offer additional benefits over hydration with half saline alone in the prevention of AKI.

Also an increased incidence of AKI with the use of i.v. sodium bicarbonate has been reported. Thus, From et al. [138] performed a retrospective study at the Mayo Clinic in Rochester, Minnesota (USA), to assess the incidence of contrast-induced AKI with the use of sodium bicarbonate and N-acetylcysteine. A total of 11,516 contrast exposures in 7977 patients had SCr values available for review before and after iodinated contrast exposure. The use of i.v. sodium bicarbonate was associated with increased incidence of contrast-induced AKI compared with no treatment.

The ERBP [115] “recommends volume expansion with either isotonic sodium chloride or sodium bicarbonate solutions, rather than no volume expansion, in patients at increased risk for AKI.”

### 5.6. Antioxidants

As mentioned, ROS have been proven to play an important role in the renal damage caused by iodinated radiocontrast agents. Hence, it is reasonable to use antioxidants for preventing AKI. Lee et al. [139] treated human embryonic kidney cells with three different contrast media: ionic HOCM ioxitalamate, nonionic LOCM iopromide, and IOCM iodixanol. All three contrast media caused a significant reduction of cell viability at 24 hours (*P* < 0.001). Short-duration pretreatment with N-acetylcysteine significantly improved cell viability compared with no N-acetylcysteine pretreatment (*P* < 0.001).

Clinical studies have suggested a protective effect of ROS scavenging with the administration of N-acetylcysteine [32, 140].

Tepel et al. [141] prospectively studied 83 patients with CRF (mean SCr of 2.4 mg/dL) planned to undergo CT with a nonionic, low-osmolality contrast agent; the 83 patients randomly received either N-acetylcysteine (600 mg orally twice daily) plus i.v. Infusion of 0.45% saline, both before and after the contrast agent (n. 41), or placebo and 0.45% saline (n. 42). An increase of at least 0.5 mg/dL of SCr 48 hours after administration of the contrast agent occurred in 1 out of 41 patients in the N-acetylcysteine group (2%) and 9 out of 42 patients in the control group (21%; *P* = 0.01). The authors concluded that N-acetylcysteine, given orally along with hydration, prevents AKI by contrast agents in patients with CRF.

Baker et al. [142] prospectively randomized 80 patients with stable renal dysfunction, planned for cardiac catheterization or intervention, to i.v. infusion of N-acetylcysteine (150 mg/kg in 500 mL normal saline, *n* = 41) or i.v. hydration alone (*n* = 39). AKI occurred in 2 out of 41 patients in the N-acetylcysteine group (5%) and in 8 out of 39 patients in the hydration group (21%; *P* = 0.045) The authors concluded that i.v. N-acetylcysteine has a protective effect against AKI.

Briguori et al. [143] tested whether a double dose of N-acetylcysteine given orally could be more effective to prevent contrast-induced AKI. They performed a prospective, randomized study on 224 consecutive patients with SCr ≥1.5 mg/dL and/or CrCl <60 mL/min, referred to their institution for coronary and/or peripheral procedures. Patients were randomly assigned to receive either 0.45% saline intravenously plus N-acetylcysteine at the standard dose of 600 mg orally twice daily (*n* = 110) or a double dose (1200 mg orally twice daily; *n* = 114) before and after nonionic, LOCM iobitridol (Xenetin-350) administration. An increase of at least 0.5 mg/dL of SCr 48 h after the procedure occurred in 12 out of 109 patients (11%) in the standard dose group and in 4 out of 114 patients (3.5%) in the double dose group (*P* = 0.038). In the subgroup with high contrast dose (≥140 mL), the AKI was significantly more frequent in the standard dose group. The authors concluded that double dose of oral N-acetylcysteine is more effective than standard dose in preventing contrast-induced AKI, particularly when high volumes of nonionic, low-osmolality contrast agent are used.

Some authors [144] demonstrated that high dose of oral N-acetylcysteine (1,200 mg twice a day before and on the day of the procedure) is more beneficial than ascorbic acid in preventing contrast-induced AKI in patients, especially in diabetic patients, with renal insufficiency undergoing coronary angiography.

Other authors did not find any significant protection by N-acetylcysteine against radiographic contrast media nephrotoxicity. Thus, Durham et al. [145] evaluated the efficacy of N-acetylcysteine for the prevention of contrast-induced AKI (defined as an increase of SCr by ≥0.5 mg/dL) in the setting of cardiac angiography: 79 patients with SCr >1.7 mg/dL were randomized to one of two groups: Group 1, i.v. hydration and N-acetylcysteine, 1200 mg 1 hour before and a second dose 3 hours after angiography; Group 2, i.v. hydration and placebo. AKI developed in 24.0% of subjects, 26.3% in the N-acetylcysteine, and 22.0% in the placebo (*P* = NS). The authors concluded that N-acetylcysteine is not effective for the prevention of AKI after cardiac angiography.

Similarly in the retrospective study of From et al. [138] at the Mayo Clinic, N-acetylcysteine alone and in combination with sodium bicarbonate was not associated with any significant difference in the incidence of contrast-induced AKI.

Allaqaband et al. [146] prospectively compared the efficacy of N-acetylcysteine, fenoldopam, and saline in preventing contrast-induced AKI in 123 high-risk patients with SCr ≥1.6 mg/dL or CrCl of <60 mL/min undergoing cardiovascular procedures. The patients received either saline (0.45% normal saline at 1 mL/kg) for 12 hours before and 12 hours after the procedure, or fenoldopam (0.1 microg/kg/min) plus saline for 4 hours prior and 4 hours after the procedure, or N-acetylcysteine orally (600 mg) plus saline every 12 hrs for 24 hours prior and 24 hours after the procedure. The authors concluded that, in patients with CRF, N-acetylcysteine or fenoldopam offered no additional benefit over hydration with saline in preventing AKI.

Goldenberg et al. [147] prospectively studied 80 patients with SCr of 2.0 mg/dL undergoing coronary angiography: patients were randomly assigned to receive either N-acetylcysteine (600 mg orally t.i.d.) or placebo, in addition to i.v. 0.45% saline (1 mL/kg/hr), 12 hrs prior to and after coronary angiography. There was no significant difference in the increase of SCr ≥0.5 mg/dL 48 hrs after coronary angiography between the N-acetylcysteine group and the placebo group (5% versus 8%, *P* = 0.52).

Similarly Coyle et al. [148] did not find any benefit by including N-acetylcysteine to the hydration regimen in patients with diabetes mellitus in preventing AKI.

Similar results have been obtained by Ferrario et al. [149] in their study in 200 elective, consecutive patients with basal CrCl ≤55 mL/min receiving either oral N-acetylcysteine (600 mg bid the day before and the day of the exposure to nonionic isosmolar contrast medium, Iodixanol, Visipaque, plus saline i.v. 0.9% 1 mL/kg/h 12–24 h before and 24 h after the procedure, *n* = 99) or placebo and saline at the same time intervals (*n* = 101). Contrast-induced AKI was 8/99 (8.1%) in the N-acetylcysteine group versus 6/101 (5.9%) in the placebo group (*P* = 0.6).

Pannu et al. [150] performed a systematic review and meta-analysis (15 studies with a total of 1776 patients) to assess the efficacy of N-acetylcysteine for preventing AKI after administration of i.v. contrast media. The authors concluded that N-acetylcysteine may reduce the incidence of acute increase of SCr after i.v. contrast administration, but this finding was of borderline statistical significance; furthermore, there was heterogeneity between trials.

Finally Gurm et al. [151] assessed the protective effect of N-acetylcysteine against AKI in consecutive patients undergoing nonemergent PCI from 2006 to 2009 in the Blue Cross Blue Shield of Michigan Cardiovascular Consortium. Of the 90,578 PCI N-acetylcysteine was used in 10,574 (11.6%) procedures. No differences in outcomes between patients treated with N-acetylcysteine and those not receiving N-acetylcysteine were observed for AKI (5.5% versus 5.5%, *P* = 0.99) or death (0.6% versus 0.8%, *P* = 0.15).

Despite these controversial results, it has been suggested to use N-acetylcysteine in high-risk patients either with an oral dose of 600 mg twice daily the day before and the day of procedure or, in patients unable to take the drug orally, with an i.v. dose of 150 mg/kg over half an hour before the procedure or 50 mg/kg administered over 4 hours [142].

Other antioxidants have been suggested to use against contrast-induced AKI: vitamin C (ascorbic acid), vitamin E (*α*- or *γ*-tocopherol), and Mesna.

Conflicting results have been obtained with the use of ascorbic acid.

Thus, some authors have demonstrated that prophylactic oral administration of ascorbic acid may protect against contrast-induced AKI [152–154].

Spargias et al. [152] conducted a randomized, double-blind, placebo-controlled trial of ascorbic acid in 231 patients with an SCr ≥1.2 mg/dL undergoing coronary angiography and/or intervention. Contrast-induced AKI occurred in 11 out of 118 patients (9%) in the ascorbic acid group and in 23 out of 113 patients (20%) in the placebo group (*P* = 0.02) thereby demonstrating a protective effect of ascorbic acid.

Alexopoulos et al. [153] examined the preventive effect of ascorbic acid on the incidence of contrast-induced AKI in 222 patients undergoing a coronary procedure. For patients who used iodixanol, the incidence of AKI was 7.4% for the ascorbic acid patients and 21.6% for placebo patients (*P* = 0.02).

Finally Sadat et al. [154] performed a systematic review with meta-analysis of randomized controlled trials (9 trials in 1,536 patients) comparing the use of ascorbic acid with placebo for the treatment of contrast-induced AKI in patients undergoing coronary angiography: patients receiving ascorbic acid had 33% less risk of AKI compared with patients receiving placebo (*P* = 0.034).

Other authors demonstrated a nonprotective effect of ascorbic acid against iodinated radiographic contrast media nephrotoxicity [155].

Thus, Boscheri et al. [155] have carried out a randomized, double-blind, prospective, and single center-study, evaluating 143 consecutive patients who received 1 g ascorbic acid or placebo plus saline hydration prior to and after angiography: no significant difference was detected in the incidence of AKI between Vitamin C patients (5/74, i.e., 6.8%) and placebo patients (3/69, i.e., 4.3%).

Tasanarong et al. [156] carried out a prospective, double-blind, randomized, and placebo-controlled trial in 305 patients with CRF undergoing coronary procedures with iopromide (LOCM). The oral administration of either 350 mg/day of *α*-tocopherol or 300 mg/day of *γ*-tocopherol (5 days prior to the procedure and continued for a further 2 days after procedure) in combination with 0.9% saline (1 mL/kg/h for 12 hours before and 12 hours after) was shown to be effective in protecting against AKI. AKI occurred in 14.9% of cases in the placebo group, but only in 4.9% and 5.9% in the *α*- and *γ*-tocopherol groups, respectively, suggesting a protective effect of vitamin E against the nephrotoxicity of iodinate contrast media.

Mesna (mercapto-ethane-sulfonate Na) is an agent with antioxidant properties that has been shown to reduce free radicals and restore reduced glutathione levels after ischemic renal failure, thereby protecting the kidneys against ischemia/reperfusion-induced oxidative damage [157]. Ludwig et al. [158] examined, in a randomized controlled trial, the efficacy of sodium 2-mercaptoethanesulfonate (MESNA), a reactive oxygen scavenger, in at-risk patients given radiographic contrast agents. The i.v. administration of 1600 mg Mesna versus placebo, together with i.v. hydration with 0.9% saline, resulted in the occurrence of AKI in 7 patients in the placebo group and none in the Mesna group (*P* = 0.005). Further studies would be necessary to confirm such a positive outcome [1].

### 5.7. Nebivolol

Nebivolol is a third-generation *β*_1_-adrenergic receptor antagonist.

Toprak et al. [159] have hypothesized that Nebivolol protects the kidney against contrast-induced AKI through its antioxidant and NO-mediated vasodilating action. In experimental Wistar-albino rats they observed that Nebivolol induced a significant increase of CrCl reduced by diatrizoate, a decrease of medullary congestion, protein casts and tubular necrosis, systemic and renal oxidative stress, microproteinuria caused by the contrast medium, and an increase of the kidney nitrite level decreased by diatrizoate.

Günebakmaz et al. [160] enrolled 120 patients undergoing coronary angiography and ventriculography, who were hydrated with i.v. isotonic saline: group I received 600 mg N-acetylcysteine every 12 hours for 4 days, group II received 5 mg nebivolol every 24 hours for 4 days, and group III patients were only hydrated: 9 patients in group I (22.5%) developed AKI, as did 8 patients (20.0%) in group II and 11 patients (27.5%) in group III (*P* = 0.72). However, a statistically significant increase in SCr was observed at day 5 compared with baseline levels only in group I (N-acetylcysteine, from 1.42 to 1.52, *P* = 0.02) and group III (hydration only, from 1.43 to 1.55, *P* = 0.01); the increase of SCr (from 1.40 to 1.48, *P* = 0.06) in group II (Nebivolol) did not reach statistical significance.

### 5.8. Statins

Recent studies have shown a beneficial effect of statins to prevent AKI in patients undergoing PCI [161–165].

Khanal et al. [162] studied 29409 patients who had both baseline preprocedure and peak postprocedure SCr measured at the time of their PCI to compare patients who received preprocedure statins with those who did not. Patients on preprocedure statins had a lower incidence of AKI (4.37 versus 5.93, *P* < 0.0001) and nephropathy requiring dialysis (0.32 versus 0.49, *P* < 0.03). They suggest initiating statin therapy before percutaneous coronary interventions.

Patti et al. [163] prospectively studied 434 patients undergoing PCI, with a follow-up for 4 years. Statin-treated patients (n. 260) had a significantly lower incidence of AKI (3% versus 27%, *P* < 0.0001) versus untreated patients (number: 174) and had better postprocedural CrCl (80 versus 65 mL/min, *P* < 0.0001); 4-year survival free of major adverse cardiac events was highest in statin-treated patients without AKI. Thus, the early protective effect of statins translates into better long-term event-free survival.

Zhang et al. [164] performed a meta-analysis of published randomized clinical trials (8 published clinical trials with 1423 patients) to evaluate whether short-term administration of high-dose statin is superior to conventional-dose statin or placebo in preventing contrast-induced AKI in patients undergoing catheterization and interventional procedures. They observed an effectiveness of short-term high-dose statin pretreatment for both decreasing the level of SCr and reducing the rate of AKI.

Current guidelines for coronary revascularization recommend the use of high dose of statins before PCI to reduce the risk of periprocedural myocardial infarction; but the beneficial clinical effect of statin pretreatment in patients undergoing coronary angioplasty arises not only from a cardiac protection against periprocedural myocardial injury but also from a renal protection against AKI caused by iodinated contrast media [165]. Actually, statins exert multiple non-lipid-lowering (pleiotropic) effects, such as improvement of endothelial function and reduction of inflammatory and immunomodulatory processes, of oxidative stress and platelet adhesion; they may contribute to both cardio- and nephroprotection even in the short-term [165].

This is not surprising, considering that hypercholesterolemia has been suggested to be a predisposing factor to ARF on the basis of a study in experimental ARF, characterized by compromised NO synthesis and enhanced ROS generation [166, 167]. But the nephroprotective effect of statins has been attributed to its antioxidant, anti-inflammatory, and antithrombotic properties and to its vasodilator property mediated by NO, which improves renal microcirculation [166, 168, 169].

Rosuvastatin (10 mg/day for five days, two days before, three days after the procedure) reduced the risk of AKI in patients with diabetes mellitus and chronic kidney disease undergoing coronary/peripheral arterial angiography [170]. Leoncini et al. [171] treated 252 patients with acute coronary syndrome, who were scheduled for an early invasive procedure and were at high risk for contrast-induced AKI, with high doses of rosuvastatin, that is, 40 mg on admission, followed by 20 mg/day. The incidence of AKI was significantly lower in the statin group than in controls (6.7% versus 15.1%, *P* = 0.003). Also simvastatin had a dose-dependent nephroprotective effect in experimental rats treated with radiocontrast agents [168]. Patients on pravastatin had an even lower incidence of AKI than patients on simvastatin [172, 173].

Acikel et al. [174] have demonstrated that short-term atorvastatin (40 mg/day 3 days before the procedure) and chronic atorvastatin therapy had a protective effect on renal function after coronary angiography.

Patti et al. [175] investigated whether short-term high-dose atorvastatin load decreases the incidence of AKI after PCI. Patients with acute coronary syndrome undergoing PCI (*n* = 241) randomly received either atorvastatin (80 mg 12 hours before intervention with another 40 mg preprocedure dose, *n* = 120) or placebo (*n* = 121): 5% of patients in the atorvastatin group developed AKI versus 13.2% of those in the placebo group (*P* = 0.046). They conclude suggesting early use of high-dose statins before percutaneous coronary revascularization to protect patients against contrast media nephrotoxicity.

### 5.9. Steroids

Ribichini et al. [176] have suggested a short course of high-dose steroids as an effective preventive measure against contrast-induced AKI: 38 patients undergoing cardiovascular procedures were given either prednisone (1 mg/kg of oral prednisone, 12–24 hours before and 24 hours after the angiographic procedure) plus i.v. saline plus hydration (1 mL/kg/hour of 0.9% saline, 12 hours before the procedure) or hydration alone. SCr was tested before and 24–48 hours after the procedure, while neutrophil gelatinase-associated lipocalin (NGAL), kidney injury molecule-1 (KIM-1), protein, and albumin were assayed in spot urine before and 6 hours after the procedure. NGAL and KIM-1 tended to rise after the procedure to a lesser degree in the prednisone group; proteinuria and albuminuria decreased significantly in the prednisone group. The authors concluded that short course of prednisone reduces the procedure-induced changes in biomarkers of renal tubular damage.

### 5.10. Diuretics

Since enhanced transport activity with oxygen consumption plays an important causal role of renal hypoxia and both furosemide and mannitol reduce transport activity, it has been suggested to use furosemide or mannitol to protect against contrast-induced AKI. Furthermore, an increase in urine output, as it occurs with furosemide and mannitol, will decrease the contact time of contrast material with tubular epithelium, thereby reducing the epithelial damage. Obviously, inducing a high urine output with diuretics in the absence of adequate fluid replacement is deleterious. Thus, the use of furosemide or mannitol should be associated with saline infusion to prevent salt depletion. Marenzi et al. [177] performed a prospective, randomized trial involving patients with CRF, defined as an eGFR less than 60 mL/min/1.73 m^2^, scheduled for coronary angiography requiring the use of the nonionic, low-osmolality contrast agent iomeprol. In their study they utilized, for prevention of AKI, the combination of hydration plus furosemide, to prevent both fluid overload in response to intravenous hydration and hypovolemia as a result of high-volume diuresis induced by furosemide administration. This was obtained by delivering intravenous fluid in an amount exactly matched to the volume of urine produced by the patient under the effect of furosemide and precisely weighed. The result was a significantly lower incidence of AKI when compared to the patients treated with only hydration.

Several studies, however, have demonstrated either no effect in protecting against contrast media or even deleterious effect of furosemide and mannitol on renal function.

Thus, Solomon et al. [178] prospectively studied 78 patients with SCr of 2.1 mg/dL undergoing cardiac angiography. Patients received either 0.45 percent saline alone for 12 hours before and 12 hours after angiography, or saline plus mannitol, or saline plus furosemide. They concluded that hydration with 0.45 percent saline provides better protection against acute decreases in renal function induced by radiocontrast agents than does hydration with saline plus mannitol or furosemide.

Similar results were obtained by Weinstein et al. [179] who concluded that furosemide may be deleterious in the prevention of radiocontrast nephropathy.

Thus, diuretics should be avoided before contrast exposure in high-risk patients who are susceptible to volume depletion.

Kurnik et al. [180] performed a multicenter, prospective, randomized, double-blind, and placebo-controlled trial to evaluate the efficacy of i.v. atrial natriuretic peptide (anaritide, ANP 4–28) to prevent contrast-induced AKI in patients with SCr >1.8 mg/dL or eGFR of ≤65 mL/min. Their conclusion was that the administration of i.v. ANP before and during a radiocontrast study did not reduce the incidence of AKI in patients with preexisting CRF, with or without diabetes mellitus.

### 5.11. Calcium Channel Blockers

Calcium Channel Blockers have been hypothesized to have protective effects against contrast-induced AKI. The rationale is the following: Ca^2+^ overload is considered to be a key factor in AKI; the increase in intracellular calcium provokes a vasoconstrictive response in intrarenal circulation and would be an important mediator of epithelial cell apoptosis and necrosis. The Na^+^/Ca^2+^ exchanger system is one of the main pathways of intracellular Ca^2+^ overload. Yang et al. [181] have demonstrated that in rats the pretreatment with KB-R7943, an inhibitor of the Na^+^/Ca^2+^ exchanger system, significantly and dose-dependently suppresses the increase of SCr following diatrizoate administration.

Thus, the use of Calcium Channel Blockers has been suggested for prevention of contrast-induced AKI. But their use has given controversial results, sometimes protective [182, 183] and sometimes with no benefit at all [178, 184].

### 5.12. Other Substances

Urinary adenosine is increased after contrast medium administration. The administration of adenosine receptor antagonists reduces the risk of development of contrast-induced AKI in both diabetic and nondiabetic patients [59]. Thus, it has been thought that adenosine antagonists (theophylline, aminophylline) could have protective effects against contrast media. But their use has given controversial results. Some authors have observed beneficial effects against AKI [185–188]; others have denied any beneficial results [189, 190].

Dopamine and dopamine agonists (e.g., fenoldopam, a selective dopamine-1 receptor agonist with vasodilatory properties) have given controversial results in protecting against CIN, some positive [191–193], others negative [146, 190, 194, 195]. On the basis of our present knowledge, it is better to avoid them, considering their adverse effects (arrhythmia with dopamine and systemic hypotension with intravenous fenoldopam).

Plasma and urine levels of endothelin-1 are increased in diabetes and after exposure to high doses of contrast media suggesting a role of endothelin-1 in diabetic nephropathy and in contrast-induced AKI [58]. However, endothelin Receptor Blockers have been proven deleterious as a prophylactic tool against contrast-induced AKI [196].

Prostaglandin E1 has given some positive protective results on renal function following contrast medium injection in patients with preexisting renal impairment [197], whilst L-arginine has shown no beneficial or even harmful effects [198].

### 5.13. Haemodialysis or Haemofiltration

It has been suggested to remove iodinated radiocontrast media by haemodialysis or haemofiltration immediately after the radiographic procedure. However, the extracorporeal removal of contrast agents did not decrease the incidence of AKI in high-risk patients [199–202]. The ERBP does “not recommend using prophylactic intermittent haemodialysis or haemofiltration for the purpose of prevention of contrast-induced AKI” [115].

## Abbreviations

AKI: Acute Kidney Injury
ARF: Acute renal failure
eGFR: estimated glomerular filtration rate
CIN: Contrast-induced nephropathy
SCr: Serum creatinine
CrCl: Creatinine clearance
CT: Computed tomography
i.v.: Intravenously
ICU: Intensive Care Unit
MDRD: Modification of Diet in Renal Disease
PCI: Percutaneous coronary interventions
NO: Nitric oxide
ROS: Reactive oxygen species
CRF: Chronic renal failure
ACEIs: Angiotensin-converting enzyme inhibitors
ARBs: Angiotensin Receptor Blockers
LOCM: Low-osmolar contrast media
HOCM: High-osmolar contrast media
IOCM: Iso-osmolar contrast media
NGAL: Neutrophil gelatinase-associated lipocalin
KIM-1: Kidney injury molecule-1.
